# Exploring the Molecular Characteristics and Role of PDGFB in Testis and Epididymis Development of Tibetan Sheep

**DOI:** 10.3390/vetsci11060266

**Published:** 2024-06-09

**Authors:** Haolin Chen, Ling Pu, Chengcheng Tian, Xingcai Qi, Juanjuan Song, Yan Liao, Bentian Mo, Taotao Li

**Affiliations:** 1 Institute of Animal Husbandry and Veterinary, Guizhou Academy of Agricultural Sciences, Guiyang 550005, China; chenhaolin612@163.com (H.C.); pulinggz2018@163.com (L.P.); 18286107320@163.com (C.T.); 13981797280@163.com (Y.L.); 2 College of Animal Science and Technology, Gansu Agricultural University, Lanzhou 730070, China; qixc@st.gsau.edu.cn (X.Q.); songjj@st.gsau.edu.cn (J.S.)

**Keywords:** PDGFB, testis, epididymis, development, sheep

## Abstract

**Simple Summary:**

Platelet-derived growth factor B (PDGFB) serves as an important cytokine for cell growth and development. Tibetan sheep (*Ovis aries*), a unique local breed in the Qinghai–Tibet Plateau, have poor reproductive physiological characteristics, including long developmental cycles, late sexual maturity, and low fecundity, yet the mechanisms behind remain elusive. Spermatogenesis and sperm maturation are highly dynamic and multifactorial developmental processes occur in the male testis and epididymis, respectively, and thus are crucial for maintaining male reproductive function and species continuity. Despite the importance of PDGFB, its role in maintaining the development and function of the testes and epididymides in sheep remains obscure. Herein, we report, for the first time, the cloning and molecular characterization of Tibetan sheep PDGFB. Meanwhile, we investigated the gene expression pattern and subcellular localization of PDGFB in developmental testes and epididymides in Tibetan sheep. The results revealed that the Tibetan sheep PDGFB gene has a high degree of evolutionary conservation and suggested that it participates in the development and function of testicular and epididymal red blood cells, Leydig cells as well as epididymal principal cells and ciliated epithelial cells, thus contributing to the spermatogenesis and sperm maturation of Tibetan sheep.

**Abstract:**

Platelet-derived growth factor B (PDGFB), as an important cellular growth factor, is widely involved in the regulation of cellular events such as cell growth, proliferation, and differentiation. Although important, the expression characteristics and biological functions in the mammalian reproductive system remain poorly understood. In this study, the PDGFB gene of Tibetan sheep was cloned by RT-PCR, and its molecular characteristics were analyzed. Subsequently, the expression of the PDGFB gene in the testes and epididymides (caput, corpus, and cauda) of Tibetan sheep at different developmental stages (3 months, 1 year, and 3 years) was examined by qRT-PCR and immunofluorescence staining. A bioinformatic analysis of the cloned sequences revealed that the CDS region of the Tibetan sheep PDGFB gene is 726 bp in length and encodes 241 amino acids with high homology to other mammals, particularly goats and antelopes. With the increase in age, PDGFB expression showed an overall trend of first decreasing and then increasing in the testis and epididymis tissues of Tibetan sheep, and the PDGFB mRNA expression at 3 months of age was extremely significantly higher than that at 1 and 3 years of age (*p* < 0.05). The PDGFB protein is mainly distributed in testicular red blood cells and Leydig cells in Tibetan sheep at all stages of development, as well as red blood cells in the blood vessel, principal cells, and the pseudostratified columnar ciliated epithelial cells of each epididymal duct epithelium. In addition, PDGFB protein expression was also detected in the spermatocytes of the 3-month-old group, spermatids of the 1-year-old group, spermatozoa and interstitial cells of the 3-year-old group, and loose connective tissue in the epididymal duct space in each developmental period. The above results suggest that the PDGFB gene, as an evolutionarily conserved gene, may play multiple roles in the development and functional maintenance of testicular cells (such as red blood cells, Leydig cells, and germ cells) and epididymal cells (such as red blood cells, principal cells, and ciliated epithelial cells) during testicular and epididymal development, which lays a foundation for the further exploration of the mechanisms by which the PDGFB gene influences spermatogenesis in Tibetan sheep.

## 1. Introduction

Tibetan sheep are a unique local sheep breed in the Qinghai–Tibet plateau and its adjacent areas which has strong adaptability to extremely poor conditions such as a high altitude, cold, hypoxia, and nutrient deficiency [1]. Tibetan sheep have reproductive physiological characteristics such as long developmental cycles, late sexual maturity, and low fecundity [2], which seriously restrict the expansion of their populations, but the underlying biological mechanisms are poorly understood. The testes and epididymides, as the core components of the male mammalian reproductive system, are the sites of spermatogenesis and sperm maturation, respectively, thus determining the male reproductive capability [3,4,5]. However, molecular biology and functional genetic studies on the reproductive system of male Tibetan sheep are still scarce.

The platelet-derived growth factor (PDGF) family, also referred to as mesenchymal growth factors, is made up of two PDGF receptor subtypes (PDGFRA and PDGFRB) and four ligands (PDGFA, PDGFB, PDGFC, and PDGFD). These subtypes interact with one another to carry out their respective biological functions [6]. PDGFs and PDGFRs are reported to be expressed and play multiple regulatory roles in the male mammalian reproductive system. For example, during the prenatal and early postnatal periods, Sertoli cells and peritubular myoid cells (PMCs) or their precursors were the primary sites of PDGF and PDGFR gene expression. These genes were also found to be expressed throughout the testes two days before birth and to increase on postnatal day five. In contrast, PDGF and PDGFR gene expression decreased to very low levels in adult testes. However, it is only expressed in Leydig cells in adult testes [7]. Since platelet-derived growth factor B (PDGFB) is primarily found in vascular endothelial cells, neurons, and megakaryocytes, its encoded protein controls cellular processes like cell division, growth, and proliferation, and is involved in numerous physiological and pathological functions [8]. The PDGFB gene has been shown to play multiple roles in gonadal development in male mice in the male reproductive system: it acts as a key migration factor of renal cells in males, whose migration is a male-specific event necessary for testicular morphogenesis; increases its testicular size by inducing testicular cell proliferation [9]. Adding PDGFB to a goat spermatogonial stem cell culture medium effectively inhibited apoptosis and promoted the self-renewal of spermatogonial stem cells. Further studies found that PDGFB could regulate phosphorylated ERK1/2 protein expression in goat SSCs through the Ras/ERK1/2 signaling pathway, thereby improving the anti-apoptotic ability of SSCs [10]. In rat and mouse epididymides, the PDGF-B gene begins to express during the first few days of life and is relatively stably expressed throughout the postnatal development involved in the morphological structure maintenance and biological function of the epididymides [11]. It can be seen that the PDGFB gene is expressed in the reproductive system of male mammals, and there are some differences in its expression and biological roles as reproductive organs develop. However, the expression profile and functional role of PDGFB in developing Tibetan sheep testes and epididymides remains unknown. Based on this, our study focused on Tibetan sheep testis and epididymis tissues at different developmental stages to reveal the sequence characteristics of the PDGFB gene and its expression and distribution in the testes and epididymides, providing a framework for the future investigation of the molecular mechanism of the PDGFB gene in the development and biological function maintenance of the reproductive system in Tibetan sheep.

## 2. Materials and Methods

### 2.1. Experimental Animals and Sample Collection

Nine healthy male Tibetan sheep from the same male parent and different female parents at three developmental stages (before sexual maturity (3 months old, *n* = 3), sexual maturity (1 year old, *n* = 3), and adulthood (3 years old, *n* = 3)), provided by the Ganjia Tibetan Sheep Breeding Cooperative in Xiahe County, Gansu Province, were used as experimental animals. All sheep lived in natural grasslands at altitudes of 3000 to 3500 m and were housed in fenced grazing with free access to feed day and night without supplementary feeding. Right testicular tissues were collected from each test sheep after slaughter, one portion fixed in 4% paraformaldehyde solution for paraffin section preparation and the other portion snap-frozen in liquid nitrogen and stored at −80 °C for RNA extraction.

### 2.2. Total RNA Extraction and cDNA Synthesis

Following a thorough grinding of the frozen tissue in liquid nitrogen, total RNA was extracted using the Trizol kit (Solaibao Technology Co., Ltd., Beijing, China). The concentration and purity of RNA were measured with a NanoDrop 2000 spectrophotometer (Thermo Fisher Scientific, Waltham, MA, USA). RNA samples with OD 260/OD 280 between 1.8 and 2.0 and OD 260/OD 230 > 2.0 were chosen, and 1% agarose gel electrophoresis was used to test for RNA integrity. Using qualified RNA samples from quality inspection as templates, first-strand cDNA was synthesized according to the operating instructions of Evo M-MLV RT Kit with gDNA Clean for qPCR Reverse Transcription Kit (Accurate Biology, Changsha, China).

### 2.3. Primer Design and Synthesis

Nucleotide sequences of PDGFB (XM_027965772.3) and β-actin (NM_001009784.3) were obtained from the NCBI database, and PCR and qPCR primers (Table 1) were designed online by Primer-BLAST and synthesized by Beijing Qingke Biotechnology Co., Ltd. (Xi’an, China).

### 2.4. Cloning of PDGFB Gene

The synthesized cDNA was used as a template for amplification of the CDS region of PDGFB. The entire reaction system for amplification was 20 μL: 2 × 10 μL of Multiplex PCR Master Mix (Kangwei Century, Taizhou, China), 1 μL of template, 1 μL of upstream and downstream primers each, and 7 μL of RNase-free water. PCR amplification program: 95 °C 5 min; 35 cycles of 95 °C 30 s, 60 °C 30 s, 72 °C 1 min; 72 °C 5 min. Following the separation of the amplified PCR products by 1% agarose gel electrophoresis, the target amplified products were recovered and purified using a gel recovery kit, subsequently linked to the pEASY-Blunt vector, and transformed to Trans5α competent cells (TransGen Biotech, Beijing, China). Seven independent positive clones were randomly selected and identified by bacterial solution PCR and sequenced by Beijing Qingke Biotechnology Co., Ltd. (Xi’an, China).

### 2.5. Bioinformatics Analysis

The cloned PDGFB sequences of Tibetan sheep were compared with existing nucleotide sequence databases in NCBI using the BLAST tool to determine the closest genetic relationship and sequence similarity, and the amino acid sequence identities of PDGFB from different species were compared using DNAMAN8.0 software. The ORFfinder online tool (https://www.ncbi.nlm.nih.gov/orffinder/, accessed on 14 December 2023) was utilized to analyze open reading frames (ORFs) within the Tibetan sheep CDS region. The physicochemical properties, functional domains, and secondary and tertiary structures of the Tibetan sheep PDGFB protein were analyzed using ProtParam (https://web.expasy.org/protparam/, accessed on 16 December 2023), InterPro (http://www.ebi.ac.uk/interpro/, accessed on 16 December 2023), SOPMA (https://npsa-prabi.ibcp.fr/cgi-bin/npsa_automat.pl?page=npsa_sopma.html, accessed on 18 December 2023), and SWISS-MODEL (https://swissmodel.expasy.org/interactive, accessed on 18 December 2023) online software. The hydrophobicity, transmembrane regions, and signal peptide areas of the protein were predicted using ProtScale (https://web.expasy.org/protscale/, accessed on 2 January 2024), TMHMM2.0 (http://www.cbs.dtu.dk/services/TMHMM-2.0, accessed on 2 January 2024), and SignalP5.0 (https://services.healthtech.dtu.dk/services/SignalP-5.0/, accessed on 2 January 2024). A phylogenetic tree of the PDGFB protein was constructed using the neighbor-joining method with MEGA7.0 software. Potential proteins interacting with the PDGFB protein of sheep were predicted and analyzed using the STRING database (https://stringdb.org, accessed on 5 April 2024), and visualized using Cytoscape 3.7.2 software.

### 2.6. qRT-PCR Analysis

With β-actin serving as an internal reference, PDGFB mRNA expression in the testes and epididymides of Tibetan sheep at different developmental stages was found via qRT-PCR as described previously [12]. A two-step reaction program was used: 95 °C for 30 s; 95 °C for 5 s, and 60 °C for 30 s for 40 cycles. The entire amplification reaction system weighed 20 μL, comprising 1 μL of cDNA, 0.4 μL of each upstream and downstream primer (Table 1), 10 uL of SYBR Green Premix Pro Taq HS qPCR Kit (Accurate Biology, Changsha, China), and 8.2 μL of RNase-free water. Each independent experiment included at least 3 replicates. Using the 2^−ΔΔCt^ method [13], the relative expression of PDGFB mRNA was determined.

### 2.7. Hematoxylin and Eosin and Immunofluorescence Staining

Hematoxylin and eosin (H&E) staining was performed to visualize the morphological changes of testes using conventional methods, as previously described [14]. Immunofluorescence staining was conducted as mentioned previously with slight modifications [15]. Briefly, the embedded tissue paraffin blocks were prepared into sections with a thickness of 4~6 μm, followed by deparaffinization, hydration, EDTA antigen retrieval, 5% BSA room-temperature blocking for 30 min, and then PBS (as negative control) and primary antibody (rabbit-derived PDGFB, 1:200; Bioss, Beijing, China), and the sections were incubated flat in a wet box overnight at 4 °C. The secondary antibody was added, incubated for 50 min at room temperature, and shielded from light after washing with PBS. Then it was washed 3 times with PBS, DAPI stainwas added, it was incubated at room temperature in the dark for 10 min, mounted with a solution containing anti-fluorescent quencher, and observed; images were acquired under the fluorescence microscope. Each independent experiment consisted of at least 3 replicates.

### 2.8. Statistical Analysis

Statistical analysis was carried out using SPSS version 21.0 statistical software (SPSS, Inc., Chicago, IL, USA). Significance was assessed using one-way ANOVA test followed by multiple comparison analysis. Data are presented as mean ± SD.

## 3. Results

### 3.1. Sequence Characteristics of Complete CDS Region of PDGFB in Tibetan Sheep

**Figure 1:**
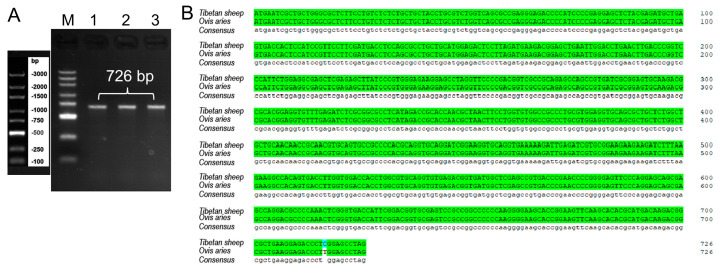
A sequencing result of PDGFB cDNA amplification product from Tibetan sheep. Note: (**A**) PDGFB cDNA amplification product. M (Lane 1): DL3000 marker; 1 (Lane 2), 2 (Lane 3), and 3 (Lane 4): samples harvested from 3−month−old, 1−year−old, and 3−year−old, respectively. (**B**) Sequence alignment between cloned and predicted PDGFB CDS region.

**Figure 2:**
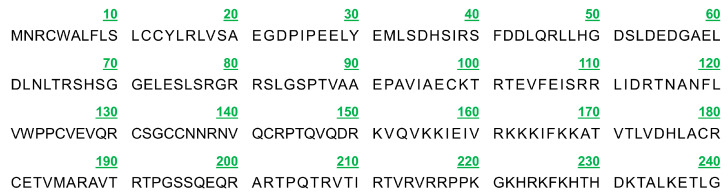
Amino acid sequence encoded by PDGFB gene in Tibetan sheep.

**Figure 3:**
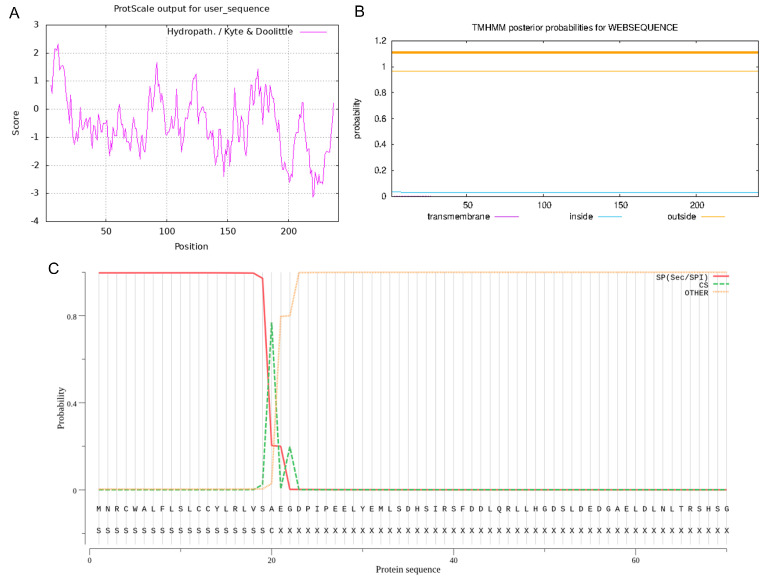
Physicochemical properties of the protein encoded by PDGFB gene in Tibetan sheep. Note: (**A**) Hydrophilic analysis; (**B**) Transmembrane structure analysis; and (**C**) Signal peptide analysis.

By using 1% agarose gel electrophoresis, the size of the PCR-amplified cDNA fragment was found to be at the anticipated location (Figure 1A). This cDNA fragment was cloned and sequenced, and a nucleotide sequence of 726 bp in length was obtained. Alignment by the NCBI online B LAST program revealed that the cloned PDGFB CDS region of Tibetan sheep had a mutation (G > A at position 10) with 99.86% similarity (Figure 1B) compared with the sheep sequence published by GenBank, but the amino acid sequence was completely consistent, which was a synonymous mutation. Four ORFs were present in this cloned sequence, of which the full-length open reading frame ORF1 (i.e., the complete CDS region) was 726 bp and could encode 241 amino acids (Figure 2). The protein encoded by PDGFB in Tibetan sheep has the following molecular formula: C 1177 H 1948 N 372 O 352 S 14. Its molecular weight is 27.39 kD, its total atomic number is 3863, and its theoretical isoelectric point (pI) is 9.54. Of the 241 amino acids in the protein, there are a total of 20 amino acid residues (Supplementary Table S1), the most abundant of which is arginine (28), accounting for 11.6% of the total content. There are a total of 29 negatively charged amino acid residues (Asp + Glu) and 43 positively charged amino acid residues (Arg + Lys). The instability index of this protein is 40.31, classifying it as an unstable protein. The aliphatic index is 80.87. The overall average hydrophobicity (GRAVY) is −0.546, indicating that it is a hydrophilic protein (Figure 3A). This protein does not contain any transmembrane regions (Figure 3B) and includes a signal peptide between the 20th and 21st amino acids (Figure 3C).

### 3.2. Homology Comparison of PDGFB between Tibetan Sheep and Other Mammals

**Figure 4:**
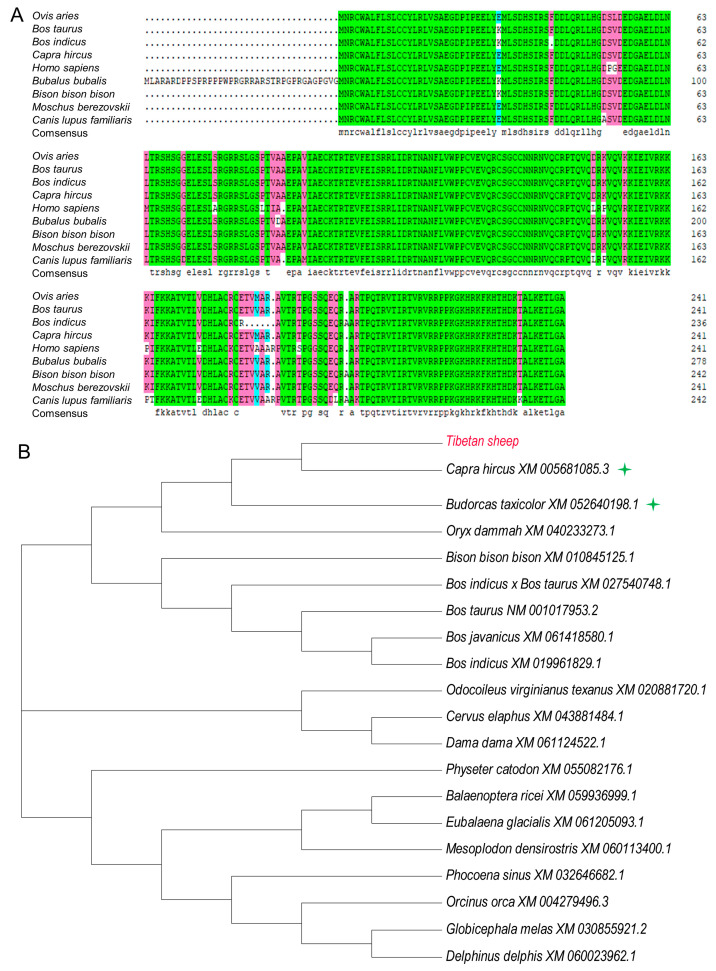
Sequence alignment of PDGFB amino acid sequences and phylogenetic tree among various species. Note: (**A**) Multiple alignment of PDGFB amino acid sequences from nine different mammalian species; (**B**) Phylogenetic tree for amino acid sequences of PDGFB protein. The closest homology with ovine DMRTC2 is indicated by four-pointed star.

Multiple comparisons of amino acid sequences of PDGFB from different mammals revealed that Tibetan sheep showed the highest similarity to their *Capra hircus* counterparts (99.59%), followed by *Moschus berezovskii* (99.17%), *Bos taurus* (98.76%), *Bison bison* (98.35%), and *Bos indicus* (95.87%) (Figure 4A). The phylogenetic tree analysis of PDGFB amino acid sequences from 20 mammalian species revealed that Tibetan sheep and goats are clustered into one branch and are evolutionarily closely related to goats and antelopes (Figure 4B).

### 3.3. Spatial Structure Analysis of PDGFB Protein in Tibetan Sheep

**Figure 5:**
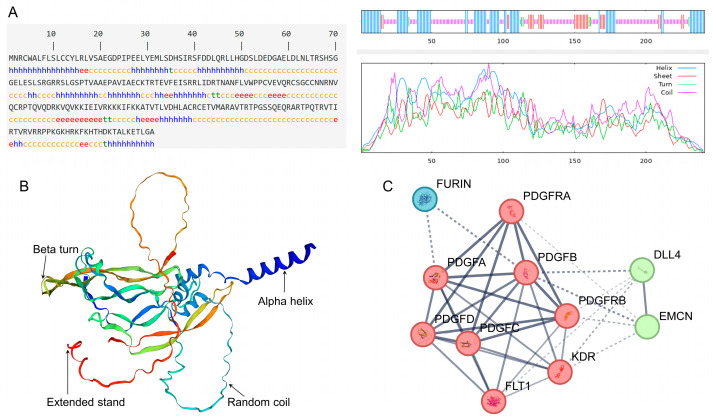
Spatial structure and interacting protein network analysis of PDGFB protein in Tibetan sheep. Note: (**A**) Secondary structure composition of Tibetan sheep PDGFB protein; (**B**) Tertiary structure prediction of Tibetan sheep PDGFB protein; (**C**) A network analysis of Tibetan sheep PDGFB protein-interacting protein.

The four structures that make up the secondary structure of the PDGFB protein found in Tibetan sheep are the α helix, β turn, random coil, and extended chain, which account for 33.20%, 2.49%, 51.87%, and 12.45% of the protein, respectively (Figure 5A). The predicted tertiary molecular structure of the PDGFB protein is similar to its secondary structure composition and mainly contains four structural types: an irregular coil, α helix, extended chain, and β turn (Figure 5B).

### 3.4. Analysis of Interacting Proteins with Tibetan Sheep PDGFB Protein

According to the results of the protein interaction network analysis, there may be interactions between the protein encoded by the PDGFB gene and 10 protein molecules in Tibetan sheep, including the congenic members PDGFA/C/D and their receptors PDGFRA/B, kinase insert domain receptor (KDR, also known as VEGFR2), FMS-like tyrosine kinase 1 (FLT1, also known as VEGFR1), Delta Like Ligand 4 (DLL4), Endothelial Mucin (EMCN), and Flynase (FURIN). PDGFB interacts with PDGFA, PDGFRA, and PDGFRB proteins with greater confidence (>0.9) (Figure 5C).

### 3.5. PDGFB mRNA Expression in Testis and Epididymis Tissues

**Figure 6:**
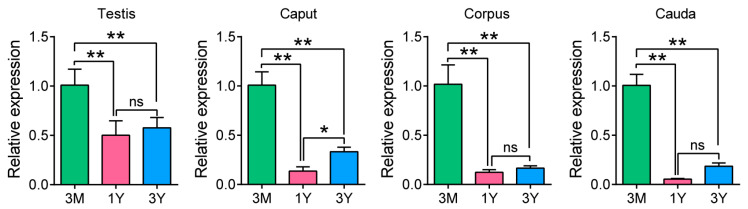
PDGFB mRNA expression in testes and epididymides of developed Tibetan sheep. Note: 3M, 3 months old; 1Y, 1 year old; 3Y, 3 years old. **, Extremely significant difference (*p* < 0.01); *, Significant difference (*p* < 0.05); ns, Non-significant difference (*p* > 0.05). Each experiment was independently repeated at least 3 times.

The QRT-PCR results showed that PDGFB mRNA expression was significantly decreased in both the testes and epididymides (head, body, and tail) of Tibetan sheep aged 1 year (after sexual maturation) and 3 years (adult) compared with 3 months (before sexual maturation) (*p* < 0.01); with the increasing age, PDGFB mRNA expression exhibited an overall trend of first decreasing and then increasing in the testes and epididymides but, this is not significant in the 1Y and 3Y groups (except for in the corpus epididymis) (Figure 6). 

### 3.6. Localization of PDGFB Protein in Testis and Epididymis Tissue

**Figure 7:**
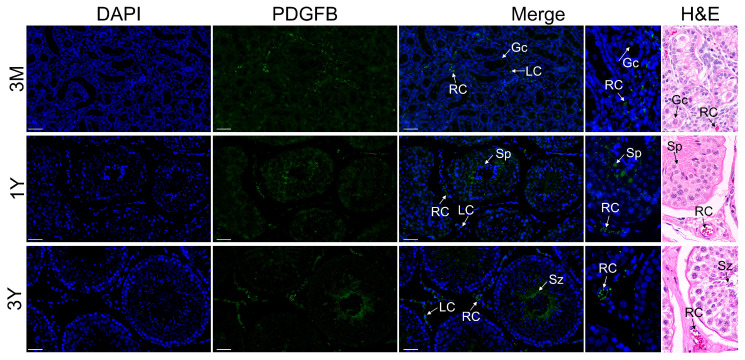
Immunofluorescence staining of PDGFB protein in testicular tissues of Tibetan sheep at different developmental stages. Note: 3M, 3 months old; 1Y, 1 year old; 3Y, 3 years old. LC, Leydig cell; Rc, red blood cell; Sp, spermatid. Scale bar, 50 um. Each experiment was independently repeated at least 3 times.

**Figure 8:**
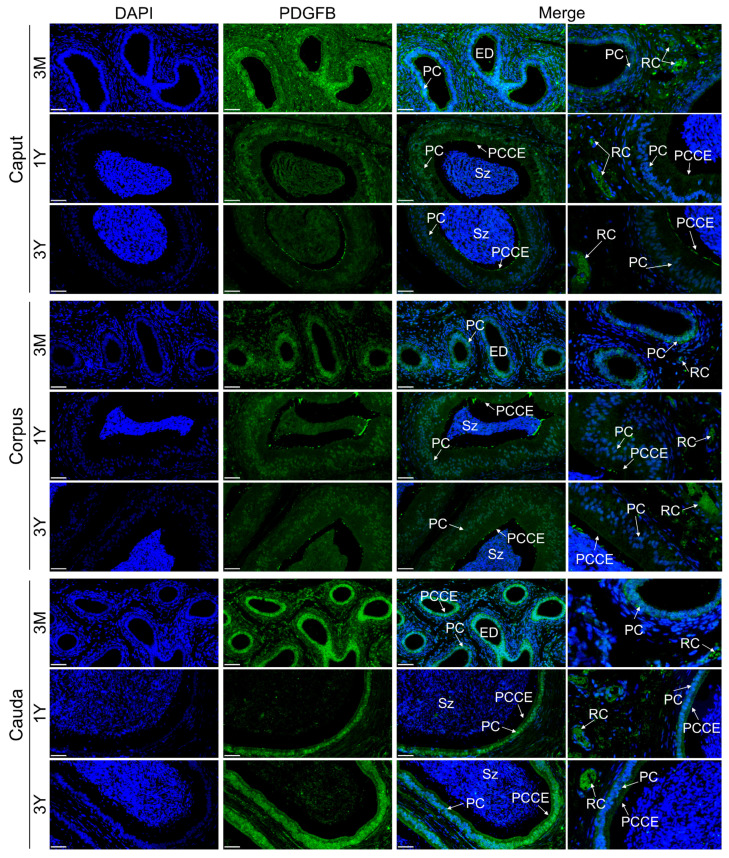
Immunofluorescence staining of PDGFB protein in each region of the epididymis of Tibetan sheep at different developmental stages. Note: 3M, 3 months old; 1Y, 1 year old; 3Y, 3 years old. PC, principal cell; PCCE, pseudostratified columnar epithelium; Sz, spermatozoon; ED, epididymal duct; RC, red blood cell. Scale bar, 50 um. Each experiment was independently repeated at least 3 times.

Immunofluorescence staining revealed that the PDGFB protein was primarily distributed in red blood cells inside the blood vessel and Leydig cells of the testes of Tibetan sheep at different developmental stages; it was also expressed in lower abundance in germ cells. In addition, there was some PDGFB protein-positive signals in the spermatocytes of the 3-month-old group, spermatids of the 1-year-old group, and spermatozoa and interstitial cells of the 3-year-old group (Figure 7). In epididymal tissues of Tibetan sheep at different developmental stages, the PDGFB protein was primarily distributed in the red blood cells in the blood vessel, principal cells of the epididymal duct epithelium and pseudostratified columnar ciliated epithelial cells in each region, and the PDGFB protein was also weakly expressed in loose connective tissue at different developmental stages in each epididymal region (Figure 8).

## 4. Discussion

In the present study, we first cloned a complete sequence of Tibetan sheep PDGFB cDNA. The PDGFB gene of Tibetan sheep, which is 726 bp long and capable of encoding 241 amino acids, was first sequenced and its entire CDS region produced by cloning. This corresponds to the PDGFB CDS region sequence (XM_027965772.3) available in NCBI at the nucleotide level, and is completely identical at the amino acid sequence level. A comparison of the amino acid sequences revealed that the amino acid sequence of the PDGFB coding region has a similarity of over 95% with *Capra hircus*, *Moschus berezovskii*, *Bos taurus*, *Bison bison bison*, and *Bos indicus*, showing the closest evolutionary relationship with goats. These results indicate that PDGFB in Tibetan sheep is highly conserved evolutionarily.

PDGF was one of the first growth factors to be characterized and is important in the development and morphological structure maintenance of various organs of animal organisms (including the testes and epididymides) in an autocrine and paracrine manner [6,16]. Rhen et al. [17] found that PDGF signaling is involved in sex determination and the differentiation of testes in crocodile turtles. In mice, PDGFB is present in developing embryonic-stage testes and induces the formation of testicular cords in organ culture [9]. PDGF signaling is required for the normal development of the Leydig cell population in adult mice, and its abnormal or absent function leads to defective Leydig cell development, smaller testes, blocked spermatocyte development, and significantly reduced sperm counts in the testes and epididymides [18]. In rats, the expression of PDGF and its receptors was tissue-specific and highly abundant in the testes [7]. PDGFB stimulates the proliferation of rat mesenchymal stem cells, while it has a significant inhibitory effect on their differentiation and testosterone synthesis [19]. Shen et al. [20] found that in developing prairie red cattle testes, the PDGF gene expression level in the testes at 1 month of age was apparently higher than that in the testes at 12, 18, and 24 months of age. In this study, our results revealed that PDGFB expression obviously decreased in the sexual maturity and adult stages compared to pre-puberty, which was generally consistent with the findings in prairie red cattle species. Interestingly, PDGFB expression showed a slightly increasing trend, perhaps in response to producing a high number of sperm in adulthood, but needs to be investigated in future studies. To further investigate the functional role of PDGFB in developing Tibetan sheep testes, the distribution of the positive signals of its encoded protein in testes at various phases of development was examined. It was found that the PDGFB protein was distributed in red blood cells inside the blood vessel, germ cells, and Leydig cells in the seminiferous tubule space of the testes of Tibetan sheep at different developmental periods. As the most abundant circulating cells in the mammalian blood, red blood cells are essential for life and metabolism, and their main role is the transportation of oxygen via the circulatory system and the delivery of oxygen to organs and cells in the body including the reproductive system [21,22,23]. Also, red blood cells participate in the transport and delivery of nutrients through the circulating system [24]. It is widely known that Leydig cells are an important component of the testicular interstitium, and their main function is to produce testosterone, which is required to guarantee normal spermatogenesis [25]. Thus, it was inferred that the PDGFB gene may be involved in the development and functional maintenance of red blood cells and Leydig cells within the testicular interstitium, thus providing required physiologic substances and hormones for contributing to testis development and spermatogenesis.

The epididymis is where sperm mature and are stored, and its morphology structure and maintenance of function are regulated by multiple growth factors, including testicular factors [26,27]. This study found that the PDGF is involved in regulating activity in wild ground squirrel epididymal cells via a paracrine/autocrine manner and in sperm maturation and storage during the breeding season [28]. Procathepsin L, which is involved in epididymal functional differentiation, is expressed and secreted in the epididymis under locally restricted conditions, and this process is directly regulated by PDGF [29]. In mice, it has been shown that the PDGFB gene starts to emerge during the first days of life and that mRNA expression is relatively stable throughout postnatal development to participate in maintaining the morphological structure and biological function of the epididymis [11]. However, currently, we have little knowledge of the PDGFB gene’s function in the development and maintenance of the epididymal in other mammals. In this study, PDGFB gene expression was found to be apparently decreased within the epididymis (caput, corpus, and cauda) of sexually mature and adult Tibetan sheep compared to pre-sexually mature, which is consistent with the findings in the testes, suggestive of potential roles in the prepubertal and adult Tibetan sheep epididymis. Subsequent subcellular localization showed that the PDGFB gene-encoded protein was primarily present in red blood cells inside the blood vessel in the interstitium around epididymal tubules as well as principal cells and pseudostratified columnar ciliated epithelial cells in each epididymal duct region segment. As the main component cells of epididymal tissue tubules, principal cells are the material basis for maintaining the physiological function of the epididymis for sperm maturation [30]. Based on the above findings, we hypothesized that the PDGFB gene may primarily be involved in the development and biological function of epididymal red blood cells, principal cells, and ciliated epithelial cells, thereby presenting a conducive microenvironment to support sperm maturation, but further research is required to determine the precise regulation. In particular, very little was reported regarding the function of testicular and epididymal vascular red blood cells at present, and therefore, further exploration on this is indeed warranted.

## 5. Conclusions

The complete CDS region of the PDGFB gene in Tibetan sheep, spanning 726 bp and encoding 241 amino acids, was successfully cloned in this study. The PDGFB gene showed an age-dependent expression profile that first decreased and then increased in the testes and epididymides of Tibetan sheep at different developmental stages, and its encoded protein was mainly present in vascular red blood cells and Leydig cells of the testes, as well as red blood cells, principal cells and pseudostratified columnar ciliated epithelial cells in various segments of the epididymides. The PDGFB gene, as an evolutionarily conserved gene, might have potential roles in the development and functional maintenance of testicular and epididymal red blood cells, Leydig cells, and epididymal principal cells and ciliated epithelial cells in Tibetan sheep for normal spermatogenesis and sperm maturation. This provides insight into further understanding the molecular mechanisms of the PDGFB gene in the maintenance of reproductive system function in male Tibetan sheep and even in other sheep breeds.

## Figures and Tables

**Table 1:** Primer Information used in this study.

Gene Name	Primer Sequences (5′~3′)	Product Length	Usage
PDGFB	F: TGAGATCGTGCGGAAGAAGAAG R: GAATGGTCACCCGAGTTTGG	161 bp	qRT-PCR
	F: ATGAATCGCTGCTGGGCG R: CTAGGCTCCAAGGGTCTCCT	726 bp	RT-PCR
β-actin	F: CTTCCAGCCTTCCTTCCTGG R: GCCAGGGCAGTGATCTCTTT	180 bp	qRT-PCR

## Data Availability

The data presented in this study are available in the article.

## Supplementary Materials

The following supporting information can be downloaded at: https://www.mdpi.com/article/10.3390/vetsci11060266/s1, Table S1: Amino acid residue composition of PDGFB gene in Tibetan sheep.
