# Supplementary material for: Exploring the Molecular Characteristics and Role of PDGFB in Testis and Epididymis Development of Tibetan Sheep
Source: Vet Sci. 2024 Jun 9;11(6):266. doi: 10.3390/vetsci11060266 (PMC11209412; doi:10.3390/vetsci11060266)
Supplement: Supplementary file 1 [file vetsci-11-00266-s001.zip › vetsci-3007585-supplementary.pdf]

Table S1. Amino acid residue composition of PDGFB gene in Tibetan sheep.

Amino acid residues	Number (piece)	Percent (%)
Ala (A)	15	6.2
Arg (R)	28	11.6
Asn (N)	7	2.9
Asp (D)	12	5.0
Cys (C)	11	4.6
Gln (Q)	9	3.7
Glu (E)	17	7.1
Gly (G)	11	4.6
His (H)	7	2.9
Ile	9	3.7
Leu (L)	23	9.5
Lys (K)	15	6.2
Met (M)	3	1.2
Phe (F)	6	2.5
Pro (P)	11	4.6
Ser	16	6.6
Thr (T)	18	7.5
Trp (W)	2	0.8
Tyr (Y)	2	0.8
Val (V)	19	7.9
